# Actinobacteria Isolated from an Underground Lake and Moonmilk Speleothem from the Biggest Conglomeratic Karstic Cave in Siberia as Sources of Novel Biologically Active Compounds

**DOI:** 10.1371/journal.pone.0149216

**Published:** 2016-02-22

**Authors:** Denis V. Axenov-Gibanov, Irina V. Voytsekhovskaya, Bogdan T. Tokovenko, Eugeniy S. Protasov, Stanislav V. Gamaiunov, Yuriy V. Rebets, Andriy N. Luzhetskyy, Maxim A. Timofeyev

**Affiliations:** 1 Irkutsk State University, Institute of Biology, Irkutsk, Russia; 2 Helmholtz Institute for Pharmaceutical Research Saarland (HIPS), Saarbrucken, Germany; 3 Universität des Saarlandes–Pharmazeutische Biotechnologie, Saarbrucken, Germany; University of Calcutta, INDIA

## Abstract

Actinobacteria isolated from unstudied ecosystems are one of the most interesting and promising sources of novel biologically active compounds. Cave ecosystems are unusual and rarely studied. Here, we report the isolation and characterization of ten new actinobacteria strains isolated from an ancient underground lake and moonmilk speleothem from the biggest conglomeratic karstic cave in Siberia with a focus on the biological activity of the obtained strains and the metabolite dereplication of one active strain. *Streptomyces* genera isolates from moonmilk speleothem demonstrated antibacterial and antifungal activities. Some of the strains were able to inhibit the growth of pathogenic *Candida albicans*.

## Introduction

Actinobacteria species are high-GC Gram-positive bacteria well-known for their prolific secondary metabolism and as a source of valuable bioactive compounds with medical and biotechnological properties [1,2]. Approximately 55% of all antibacterial compounds come from the *Streptomyces* genera, and another 11% are derived from other actinobacteria [3]. Identification of new antibiotic agents is important due to the emergence of new pathogens and antibiotic resistance [4]. In this regard, the actinobacteria isolated from unusual ecosystems are one of the most interesting sources of novel biologically active compounds [5, 6].

Cave ecosystems are rarely studied and contain different mineral formations, permafrost and previously unknown organisms that have evolved microclimates with minor variations in temperature, humidity, air composition and other parameters over extended periods of time [7]. Whether specific substances, especially moonmilk speleothem, exist in some caves is unclear and has been discussed in previous studies [8, 9]. As described in Encyclopedia of Geobiology (2011) the moonmilk is a carbonate deposit that occurs within various subterranean systems. Moonmilk has a white to gray color and, in contrast to rigid cave deposits such as stalactites and stalagmites, exhibits a soft, muddy texture of microcrystalline aggregates. These aggregates are mainly composed of calcite, and to a lesser extent of aragonite, monohydrocalcite, hydromagnesite, sulfates, and nitrates [10].

Cave ecosystems are of great scientific interest due to the development of the macro and microorganisms, which have undergone long evolutions in stable conditions. In addition, caves contain limited materials and have little energy exchange with the environment [11–14].

Knowledge regarding cave microbial and especially actinobacterial diversities is very limited, despite the fact that numerous caves have been identified throughout the world [12]. The microbial diversity of karstic caves has been studied to understand the impacts of microbes on cave formation and to determine how this knowledge can be used for cave conservation [15–21].

M. Maciejewska and coauthors (2015) reported the isolation of the strain *Streptomyces lunaelactis* sp. nov. from moonmilk speleothem collected in the cave ‘Grotte des Collemboles’(Comblain-au-Pont, Belgium) [9]. Jurado and coauthors (2010) reported the isolation of 34 novel species of actinobacteria from subterranean environments [22]. These novel actinobacteria were exemplified in the descriptions of *Beutenbergia cavernae*, a new genus of L-lysine-containing actinobacteria, and *Agromyces subbeticus*, isolated from a cave in southern Spain [23–24].

We suggested that microorganisms inhabiting an isolated ecosystem of the caves, such as biggest conglomeratic karstic cave in Siberia, should preserve a high number of unstudied bacterial strains with specific metabolic pathways and secondary products. Their isolated evolution increases the expectation that secondary metabolism genes evolved separately from the general pool and elevates the likeliness to find novel biologically active compounds.

The main goal of the current research was to isolate new actinobacteria strains from atypical sources such as the water surface from an ancient underground lake and moonmilk speleothem from the Bolshaya Oreshnaya cave (Siberia) to estimate the antibiological activity with the following dereplication of secondary metabolites.

## Materials and Methods

### Cave description and sampling

The Bolshaya Oreshnaya cave (55° 17′ 34.8″ N, 93° 44′ 8.52″ E) is one of the biggest conglomeratic karstic caves in the world [25]. The cave is located in the Mansk area of the Krasnoyarsk region and contains a federally ranked geomorphological natural monument (Siberian Federal Distinct, Siberia, Russia). The Mansk area is one of the most interesting karstic and speleologic areas in the region. This area contains 450 million-year-old Ordovic basal sediment conglomerates that are nearly two-kilometers thick. The Bolshaya Oreshnaya cave is unique due to its age, size and karst cavity structural features. It ranks first in the world in length cavity among karst formations encountered in these rocks. The map of the Bolshaya Oreshnaya cave is available [26]. There are approximately 20 grots connected by an extensive system of tunnels and several lakes up to 15 m deep within the cave. Thus, there are numerous lake wells, balustrades, columns, protected areas with almost transparent stalactites, stalagmites, and draperies in the cave. However, the cave has not yet been fully explored, especially by those in the microbiology and biotechnology fields. Currently, the known depth of the cave is 195 meters, amplitude 247 meters and length of the known passages 47–58 km. The age of the cave is approximately 20–25 million years, and the annual temperature is very stable and fluctuates approximately 3°C [25, 27].

The water from the underground lake and the moonmilk speleothem of split Kolokolniy from Bolshaya Oreshnaya cave were chosen as sources of actinobacteria strains. Water surface and moonmilk samples were collected by sterile syringes and spatulas in sterile tubes, respectively. Several replicates (3–5) of each sample were collected in November 2014. Then, the samples were transferred to the laboratory in thermostatic conditions (3–5°C) where they were stirred intensively in 20% sterile glycerol at a ratio of approximately 1:10 and stored at -20°C.

### Actinobacteria isolation

The isolation of actinobacteria strains was performed on the solid nutrient media MS (soy flour– 20 g/L; D-mannitol– 20 g/L; agar– 20 g/L; pH 7.2) and agarised DNPM (DNPM^A^) (soytone– 7.5 g/L; baker yeast– 5 g/L; MOPS– 21 g/L; dextrin—40 g/L; agar– 20 g/L; pH 6.8)) supplemented with the antibiotics cycloheximide (50 μg/mL) and phosphomycin (100 μg/mL) [28]. At the same time, sample aliquots were preheated for 5 minutes at 50°C to activate the spores. All samples were diluted in a 1% sterile saline solution at 1:10, 1:100 and 1:1000 ratios, and the dilutions were plated on the MS and DNPM^A^ media in 3 replicates. Plates were incubated for 30 days at 28°C and checked every 24 hours for actinobacterial colony appearance. Actinobacteria-like strains were identified based on colony morphology [28]. Colonies were transferred from the primary plates onto new MS media. An attempt was made to obtain pure cultures of all colonies observed on the primary plates. Active and rare strains were deposited in the Russian Collection of Agricultural Microorganisms (RCAM), St. Petersburg. Information about the strain deposition is available at www.arriam.spb.ru. The accession numbers of the deposited strains are presented in Table 1.

**Table 1:** Actinobactreria strains isolated from water from an underground lake and moonmilk speleothem from the Bolshaya oreshnaya cave.

Source	Actinobacterial isolates	Query score, %	Sequence ID	Closest homologue strains, Name and Sequense ID
Moonmilk speleothem	*Streptomyces* sp. IB 2014/I/78-8 (RCAM ID 03472)	100	KT266894	*Streptomyces parvus* 259–8|KT191167; *Streptomyces badius* 89| KT191165; *Streptomyces griseus* 44(1)| KT191164
Moonmilk speleothem	*Streptomyces* sp. IB 2014/I/78-1	100	KT266895	*Streptomyces* sp. ASM211| JN866676; *Streptomyces californicus* A-J15-2-4| EU570722; *Streptomyces cinereorectus* 6–7| KJ571053
Moonmilk speleothem	*Nocardia* sp. IB 2014/I/79-1HS	100	KT266896	*Nocardia cummidelens* HBUM174688| FJ532399; *Nocardia* sp. QLS42| JQ838093;*Nocardia fluminea* | NR117325
Moonmilk speleothem	*Streptomyces* sp. IB 2014/I/78-12	100	KT266897	*Streptomyces* sp. YC-8| KJ139433; *Streptomyces anulatus* AMU11| JN652249; *Streptomyces fimicarius* 14–5| KJ571079
Moonmilk speleothem	*Streptomyces* sp. IB 2014/I/78-3	100	KT266898	*Streptomyces* sp. QLS13| JQ838123; *Streptomyces argenteolus* AB907697; *Streptomyces sporovirgulis* TGNBSA5| JQ654447
Moonmilk speleothem	*Streptomyces* sp. IB 2014/I/78-6	100	KT266899	*Streptomyces pulveraceus* HHI2| KJ573063; *Streptomyces scopuliridis* 3H8| KF600747; *Streptomyces nodosus* XSD-120| EU273535
Moonmilk speleothem	*Streptomyces* sp. IB 2014/I/78-11	100	KT266900	*Streptomyces* sp. A132| JN866692; *Streptomyces* sp. 13(2014)| KF772614; *Streptomyces olivochromogenes* xsd08157| FJ481073
Moonmilk speleothem	*Streptomyces* sp. IB 2014/I/78-9	100	KT266901	*Streptomyces* sp. BA11| EF494206; *Streptomyces pratensis* XJB-YJ17| KM186629; *Streptomyces badius* CB00830 | HF935087
Water of underground lake	*Nocardia* sp. IB 2014/I /100-5 (RCAM ID 03475)	100	KT266902	*Nocardia* sp. DSM 6249| AF430063; *Nocardia* sp. R26| AF277216; *Nocardia cummidelens* 174458| EU593717
Water of underground lake	*Nocardia* sp. IB 2014/I /100-1HS (RCAM ID 03474)	100	KT266903	*Nocardia fluminea* 173590| EU593589; *Nocardia salmonicida* 173685| EU570345; *Nocardia* sp. SXYM10| GQ357986

RCAM—Russian Collection of Agricultural Microorganisms (RCAM), St. Petersburg. Information about the strain deposition is available at www.arriam.spb.ru.

### 16S rRNA gene sequencing and analysis

Strains were grown in 10 mL of TSB media at 28°C for 3 days at 180 rpm and total DNA was isolated as described [28]. Amplification of the 16S rRNA gene was carried out with the following primers: 8F (AGA GTT TGA TYM TGG CTC AG) and 1510R (TAC GGY TAC CTT GTT ACG ACT T). The obtained fragments were gel purified using the QIAquick Gel Extraction Kit (Qiagen, Venlo, Netherlands) and sequenced with the amplification primers 8F and 1510R to generate a nearly complete gene sequence (1351~1396 bp). The forward and reverse sequences were assembled with SeqMan DNAStar software (Lasergen, Houston, USA). 39 sequences were multiple-aligned using MAFFT v7.017 (algorithm: auto, gap open penalty: 1.53, offset value: 0.123). The dendrogram was constructed using RAxML 7.2.8 (nucleotide model: GTR GAMMA, algorithm: bootstrapping, bootstrap replicates: 1000), and formatted using Geneious 8.1.7 [29–31]. *E*. *coli* as an outgroup. The sequences were deposited in the GenBank (accession numbers: KT266894-KT266903).

### Metabolites analysis

#### Cultivation and extraction

Isolated strains were inoculated in 10 mL of TSB media, grown for 2 days at 28°C at 180 rpm, and 2 mL of pre-culture were used to inoculate the 30 mL of production media in a 250 mL-Erlenmeyer flask with baffles. Two different liquid media (NL19 and DNPM) were chosen for metabolite production [28] because the media composition is often a determining factor for the production of different secondary metabolites. The media compositions were as follow: NL-19 (soy flour– 20 g/L; D-mannitol– 20 g/L; pH 7.2) and DNPM (soytone– 7.5 g/L; baker yeast– 5 g/L; MOPS– 21 g/L; dextrin—40 g/L; pH 6.8). The strains were cultivated on both types of media at 28°C at 180 rpm for 4 days. The liquid cultures and biomass were separated by centrifugation at 3000 rpm for 5 min. Metabolites from the liquid cultures were extracted with ethyl acetate (Sigma, St. Louis, USA) (ratio1:1). The compounds from the biomass were extracted with an acetone:methanol mixture (ratio 1:1) as described in [32].

Pure actinobacteria cultures grown on solid nutrient media (MS and DNPM^A^) were homogenized, and metabolites were extracted with an acetone:methanol mixture as described above for biomass compounds. The extracts were evaporated in rotary evaporator IKA RV–8 at 40°C and dissolved in 500 μL of methanol. For screening of active strain all cultivation and extraction procedures were done three times.

#### High performance liquid chromatography separation

The extract of one active strain (*Streptomyces* sp. IB2014/78-8, S1 Fig) was cultivated in 1.5 L media of DNPM. The secondary metabolites from the liquid culture were extracted by ethyl acetate as described above. Then, the extract was initially (per minute) separated on an Ultimate 3000 HPLC system (Dionex, Sunnyvale, USA) using a C18 column (Affymetrix, Santa Clara, USA), and a linear gradient of acetonitrile against 0.1% ammonium formate solution in water was flowed over the column at 5 mL/min for 23 minutes. Thus, 23 fractions of separated extract were prepared. The antimicrobial activities of the fractions were analyzed against those of the bacteria described in the paragraph “Biological activity assay of extracts from isolated strains”.

#### Liquid chromatography-mass spectrometry (LC-MS) analysis

The active fractions obtained were analyzed by ultra-high resolution mass spectrometry using an LC-QTOF system maXis II (Bruker, Billerica, USA). The samples were separated on an Ultimate 3000 HPLC system (Dionex, Sunnyvale, USA) using a C18 column (Affymetrix, Santa Clara, USA) and a linear gradient of acetonitrile against 0.1% ammonium formate solution in water at a flow rate 0.5 mL/min for 20 min. The mass detection was performed in both positive and negative modes with the detection range set to 160–2500 m/z. Data were collected and analyzed by Brucker Compass Data Analysis software, version 4.1 (Bruker, Billerica, USA). The screening for known compounds was performed using the Dictionary of Natural Products database version 6.1 (CRC Press, Boca Raton, USA) with the following search parameters: accurate molecular mass, absorption spectra and source of compound isolation [33]. Compounds were considered to be similar when the difference in accurate mass was close to 0.01 and the absorption spectra were identical. This part of the study was completed at Helmholtz-Institut für Pharmazeutische Forschung Saarland (HIPS).

### Antibiological activity assay of extracts from isolated strains

Several types of antibiological activity were investigated. Antibacterial and antifungal activities were estimated for all strains and all type of extracts. The antimicrobial activities of the extracted metabolites were assayed by disk diffusion method [34]. We used this method for qualitative assessment of antimicrobial activities and for primary screening of active strain. For this approach, 40 or 25 μl of each extract was loaded on 6- or 4-mm diameter paper discs, respectively. Test cultures of *Bacillus subtilis* ATCC 6633*7*, *Pseudomonas putida* KT 2440, and *Escherichia coli* ATCC25922, were plated from the liquid cultures on LB broth dried for 20 minutes prior to disc loading. The antifungal activity of each sample was tested against that of *Saccharomyces cerevisiae* BY4742, *Candida albicans* DSM1665 and *Fusarium verticillioides* DS*M 62264*. The media used for fungus growth was YPD (yeast).

The test cultures were obtained from the Leibniz-Institut DSMZ-German Collection of Microorganisms and Cell Cultures (Braunschweig, Germany). The activity against *C*. *albicans* was estimated in Microbial Natural Products at HIPS. The inhibiting influence of extracts on *F*. *verticillioides* was estimated in Irkutsk regional veterinary laboratory. The zones of inhibition were measured manually with accuracy ±1 mm after overnight cultivation.

## Results and Discussion

### Isolation and phylogenetic characterization of actinobacteria from the underground lake and moonmilk speleothem

Initial screening provided ten independent isolates based on morphological features. As can be observed from Table 1, the majority of isolates (eight out of ten) were obtained from samples that originated from moonmilk speleothem, and two strains were isolated from the water of the underground lake.

**Fig 1:**
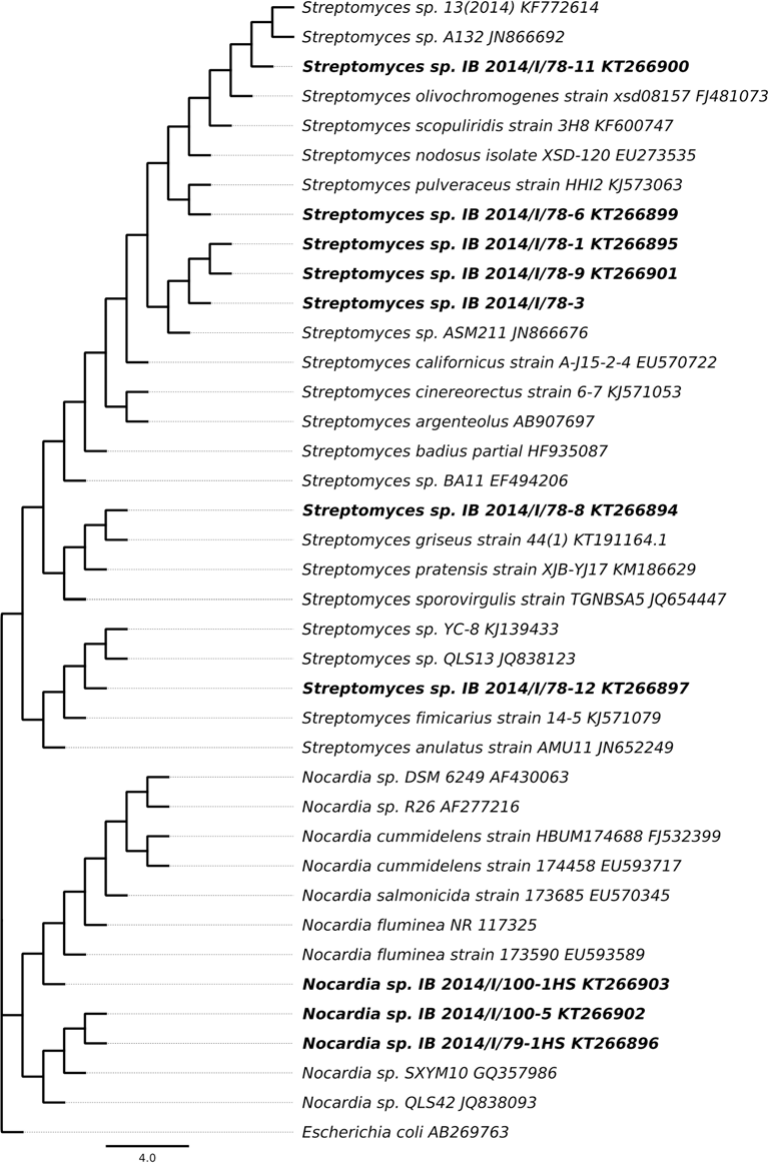
Phylogenetic tree of actinobacteria isolates based on the 16S rRNA gene sequences.

16S rRNA gene analysis based phylogenetic analysis of the obtained strains showed that all strains isolated from the water belong to the genus *Nocardia*. Seven out of eight actinobacterial strains isolated from moonmilk speleothem belong to *Streptomyces* and one to the *Nocardia* genera. 16S rRNA form a tight clade with several representatives of respective genera (Fig 1). This finding indicates the diversity of actinobacteria species that could be isolated with this approach.

### Analysis of biological activity of the isolated strains

Five strains cultivated on DNPM and six strains cultivated on NL-19 media were active against Gram-positive bacteria *B*. *subtilis* (Table 2; S1 Table, S2 Table). After growth on the NL-19 media, the strain *Streptomyces* sp. IB 2014/I/78-8 was no longer active against all bacterial strains, including *B*. *subtilis*. However, strains *Streptomyces* sp. IB 2014/I/78-12 and *Streptomyces* sp. IB 2014/I/78-11 that did not inhibit the growth of *B*. *subtilis* when cultured on the DNPM media inhibited *B*. *subtilis* after growth on NL-19 media.

**Table 2:** Antimicrobial activity of isolated strains when grown on different mediums.

Strain	DNPM/DNPM^A^ media			NL-19/MS media		
	Biomass extract	Cultural extract	Biomass-agar extract	Biomass extract	Cultural extract	Biomass-agar extract
***Streptomyces sp*. *IB 2014 /I/78-8***	-	Bs, Pp, Ec, Са	-	-	-	-
***Streptomyces sp*. *IB 2014 /I/78-1***	Bs	-	-	Bs	-	-
***Nocardia sp*. *IB 2014 /I/79-1HS***	-	-	-	-	-	-
***Streptomyces sp*.*IB 2014 /I/78-12***	-	-	-	-	-	Bs
***Streptomyces sp*. *IB 2014 /I/78-3***	Fv	Bs, Fv	Bs, Sc, Ca, Fv	Bs, Fv	Bs	Bs, Fv
***Streptomyces sp*. *IB 2014 /I/78-6***	-	-	Bs	Bs,Ec	-	Bs
***Streptomyces sp*. *IB 2014 /I/78-11***	-	-	-	-	-	Bs
***Streptomyces sp*. *IB 2014 /I/78-9***	Bs, Fv	-	-	Bs	-	-
***Nocardia sp*. *IB 2014 /I/100-5***	-	-	-	-	-	-
***Nocardia sp*. *IB 2014 /I/100-1HS***	-	-	-	-	-	-

Bs—B. subtilis; Pp—P. putida; Ec—E. coli; Ca—C. albicans; Sc–S. cerevisae; Fv–F. verticillioides

Strain *Streptomyces* sp. IB 2014/I/78-8 cultivated on DNPM and strain *Streptomyces* sp. IB 2014/I/78-6 cultivated on NL-19 medium were active against Gram-negative bacteria (Table 2; S1 Table, S2 Table). The extract obtained from the liquid culture of strain *Streptomyces* sp. IB 2014/I/78-8 inhibited the growth of both *E*. *coli* and *P*. *putida*. The strain *Streptomyces* sp. IB 2014/I/78-6 inhibited the growth of *E*. *coli* but not of *P*. *putida* by compounds extracted from the strain biomass.

Several strains demonstrated activity against fungus after cultivation on DNPM media. Thus, two strains, *Streptomyces* sp. IB 2014/I/78-8 and *Streptomyces* sp. IB 2014/I/78-3, were active against *C*. *albicans*. Strain *Streptomyces* sp. IB 2014/I/78-3 was characterized by large antifungal activity in all type of extracts and inhibited the growth of other fungi such as *S*. *cerevisiae* and *F*. *verticillioides*. *Streptomyces* sp. IB 2014/I/78-9 also demonstrated activity against *F*. *verticillioides* (Table 2; S1 Table, S2 Table).

After cultivation on NL-19 media, the strain *Streptomyces* sp. IB 2014/I/78-3 presented activity against *F*. *verticillioides*. The other strains did not show antifungal activity after growing on NL-19 media.

Thus, the *Streptomycetes* spp genera strains isolated from moonmilk speleothem demonstrated antibacterial and antifungal activities. In general, the observed bioactivity of crude extracts of isolated strains broadly consistent to bioactivity of a crude extracts obtained from other published bacterial isolates [35–36] but couldn’t be compared with purified known commercial antibiotics. The materials about activity of some known antibiotics available online in [Clinical Laboratory Improvement Amendments, https://www.cms.gov/Regulations-and-Guidance/Legislation/CLIA/index.html?redirect=/clia; https://www.cms.gov/regulations-and-guidance/legislation/clia/downloads/sc04163.pdf].

Observed differences in the bioactivity of strains cultivated on the different mediums and bioactivity of crude extracts obtained from culture liquid and the cell biomass are caused by the exocellular and intracellular synthesis of compounds and their physical and chemical characteristics [28].

### HPLC separation and dereplication of secondary metabolites profile of active strain *Streptomyces* sp. IB 2014/I/78-8

The majority of pathogenic Gram-negative bacteria are inadequately controlled by drugs. Thus, strains producing metabolites capable of inhibiting the growth of *E*. *coli* and *P*. *putida* in our tests are of particular interest. We conducted dereplication analyses of the metabolic profiles of active fractions obtained from separated extract of the culture liquid of strain *Streptomyces* sp. IB 2014/I/78-8.

Table 3 and S3 Table display results characteristic of antimicrobial activity. Thus, six fractions out of 23 were active against Gram-positive bacteria, and four fractions were active against Gram-negative bacteria. Five of the fractions inhibited the growth of the pathogenic yeast *C*. *albicans* but not of *S*. *cerevisiae*.

**Table 3:** Antimicrobial activity in separated fraction extract obtained from liquid culture of strain *Streptomyces sp*. *IB 2014/I/78-8* grown on DNPM media.

Test culture	Fraction, Min / Activity																						
	1	2	3	4	5	6	7	8	9	10	11	12	13	14	15	16	17	18	19	20	21	22	23
***B*. *subtilis***	-	+*	-	-	-	+*	-	-	-	-	+*	+	-	-	+	-	+	-	-	-	-	-	-
***E*. *coli***	-	-	-	-	-	-	-	-	-	-	-	+*	-	-	+*	-	-	-	-	-	-	-	-
***P*. *putida***	-	+	-	-	-	+	-	-	-	-	-	-	-	-	-	-	-	-	-	-	-	-	-
***C*. *albicans***	-	-	-	-	-	-	-	-	-	-	-	-	-	-	-	-	-	-	+*	+*	+*	+*	+*
***S*. *cerevisae***	-	-	-	-	-	-	-	-	-	-	-	-	-	-	-	-	-	-	-	-	-	-	-

*—Bacteriostatic activity

Modern mass spectrometry methods allow not only the general analysis of secondary metabolites but also the possibility for identification of individual compounds using information from pre-existing databases. There were 122 compounds detected in the active fractions (S4 Table). Twenty compounds could be preliminarily predicted based on characteristics from the Dictionary of Natural Products database using search parameters described in the method section. A total of 102 compounds could not be predicted and appear to be novel. Only actinobacterial compounds and compounds with molecular weight higher that 400m/z are described below.

**Fig 2:**
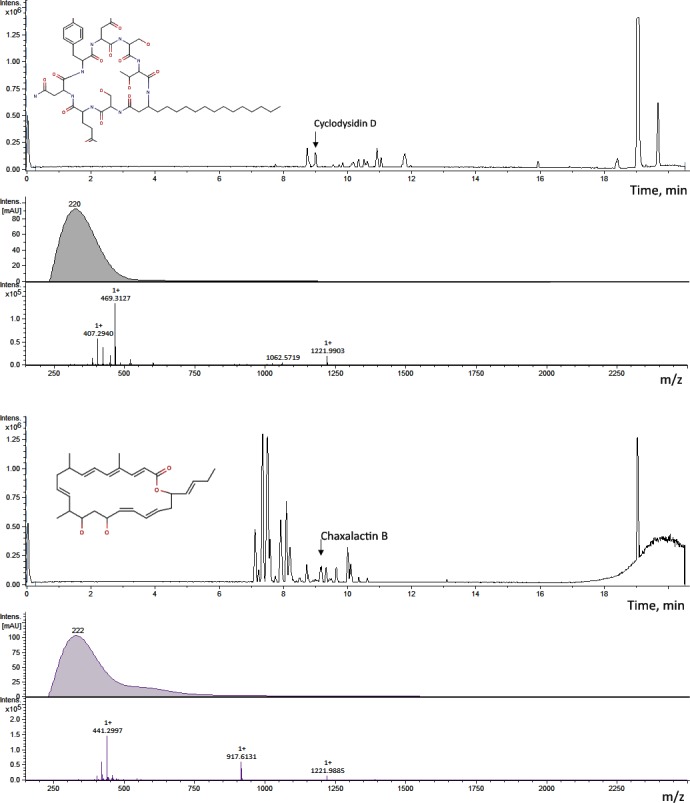
Structure of Cyclodysidin D and Chaxalactin B and their identification in extract of *Streptomyces sp*. *IB 2014/I/78-8*.

Isolate *Streptomyces* sp. IB 2014/I/78-8 was found to produce two *Streptomyces* genera compounds: Cyclodysidin D (CRC-number QQS86-P) and Chaxalactin B (CRC-number QMD43-B) (Table 4, Fig 2). These compounds were found in fractions collected at 18^th^ and 15^th^ minutes of extract separation, respectively. As previously stated, the Cyclodysidin D produced by *Streptomyces* sp. RV 15 is associated with the marine sponge *Dysea tupha*. The current compound is a cyclic lipopeptide [37]. The antibiotic role of this compound is not well-described. At the same time, the high antibiotic activity of the Chaxalactin B compound produced by *Streptomyces* sp. C 34 has been described in relation to Gram-positive bacteria [38]. Chaxalactin B is related to ansamycin-type polyketides. Strain *Streptomyces* sp. C 34 was isolated from a hyper-arid soil samples collected from the Chaxa de Laguna, Salar de Atacama of the Atacama Desert [38, 39]. Busarakam et al. (2014) proposed the name for this taxon as *Streptomyces leeuwenhoekii* sp. nov. in which strain C34T possesses a remarkably large number of gene clusters involved in natural product synthesis [40].

**Table 4:** Some compounds identified with DNP from extracts of isolated strain *Streptomyces sp*. *IB 2014/I/78-8*.

Compound name	Cyclodysidin D	Chaxalactin B, 14-Deoxy	Stylissazole B	Gyrophoric acid (4-Me ether, L-alanine amide)
**Dictionary of natural products:**				
**CRC-number**	QQS86-P	QMD43-B	QGS30-C	NPB34-D
**Calculated Mass**	1061.575714	440.29266	554.133796	553.158414
**Molecular Formula**	C49H79N11O15	C28H40O4	C22H23BrN10O3	C28H27NO11
**UV maxima**	224 (MeOH)	229 (MeOH)	220 (MeOH)	216,266,305
**Biological Source**	*Streptomyces sp*. *RV15*	*Streptomyces sp*. *C34*	*Stylissa carteri*	*Humicola sp*. *FO-2942*
**Experimental data:**				
**Detected mass, m/z**	1062.5729	441.2997	555.1504	554.18
**Accurate mass**	1061.565	440.2917	554.1424	553.172
**UV maxima, nm**	220	222	214	220, 270,300
**Δ Accurate mass**	0,01	0,001	0,01	0,01

According to preliminary results, other compounds found in extracts of the strain *Streptomyces* sp. IB 2014/I/78-8 were related to pyrrole-2-aminoimidazole alkaloid Stylissazole B (Stylissazole B, CRC-number QGS30-C), which was previously isolated from the marine sponge *Stylissa carteri* [41], and Gyrophoric acid (4-Me ether, L-alanine amide, CRC-number–NPB34-D), which is a known inhibitor of diacylglycerol acyltransferase and an antilipaemic agent [42]. Several additional compounds were preliminary predicted in extracts of the strain *Streptomyces* sp. IB 2014/I/78-8. However, their biological importance is not clear (S4 Table).

The medical properties of moonmilk speleothem have been described in medical treatises. It is known that moonmilk was used to heal bone fracture and chancre. Thus, moonmilk was used as a drug for diarrhea and dysentery. In veterinary medicine, moonmilk was used as a drug for cows with reduced lactation [43]. However, the definite effects of the drug as well as the geochemical composition of moonmilk and antibiotic compounds produced by actinobacteria remain unknown [25].

As a result, we have isolated ten actinobacteria strains from the moonmilk speleothem and an underground lake located in one of the biggest conglomeratic karstic caves in the world. *Streptomyces* genera isolates from moonmilk speleothem demonstrated antibacterial and fungicidal activities. Several fractions of the strain *Streptomyces* sp. IB 2014/I/78-8 and crude extract of *Streptomyces* sp. IB 2014 /I/78-3 were able to inhibit the growth of pathogenic *C*. *albicans*. These strains represent a particular interest for enlarged investigation, including isolation and characterization of pure active compounds and study of their biosynthesis. In conclusion, we can postulate that the majority of secondary metabolites, including antibacterial and fungicidal compounds synthesized by the isolated strains are not described in the huge available database “Dictionary of Natural products” (CRC-press), which includes more than 272 thousands compounds. This fact confirms that the chosen strategy for isolation of new actinobacteria from these unique ecological niches, such as formations of ancient and isolated caves’ ecosystems could be successful and effective to found new biologically active compounds.

## Supporting Information

S1 Fig Photo of strain *Streptomyces sp*. *IB 2014/I/78-8*. (PDF)

S1 Table The antimicrobial activity of biomass and culture fluid extracts of cultured actinobacteria strains grown in DNPM/ DNPM^A^ media. (PDF)

S2 Table The antimicrobial activity of biomass and culture fluid extracts of cultured actinobacteria strains grown in NL-19/ MS media. (PDF)

S3 Table The antimicrobial activity of biomass and culture fluid extracts of cultured actinobacteria strains grown in NL-19/ MS media. (PDF)

S4 Table Number and characteristics of main peaks and compounds produced by *Streptomyces sp*. *IB 2014 /I /78-8*. (PDF)
