# Correction: Actinobacteria Isolated from an Underground Lake and Moonmilk Speleothem from the Biggest Conglomeratic Karstic Cave in Siberia as Sources of Novel Biologically Active Compounds

**DOI:** 10.1371/journal.pone.0152957

**Published:** 2016-03-30

**Authors:** Denis V. Axenov-Gribanov, Irina V. Voytsekhovskaya, Bogdan T. Tokovenko, Eugeniy S. Protasov, Stanislav V. Gamaiunov, Yuriy V. Rebets, Andriy N. Luzhetskyy, Maxim A. Timofeyev

The first author’s name is spelled incorrectly. The correct name is: Denis V. Axenov-Gribanov. The correct citation is: Axenov-Gribanov DV, Voytsekhovskaya IV, Tokovenko BT, Protasov ES, Gamaiunov SV, Rebets YV, et al. (2016) Actinobacteria Isolated from an Underground Lake and Moonmilk Speleothem from the Biggest Conglomeratic Karstic Cave in Siberia as Sources of Novel Biologically Active Compounds. PLoS ONE 11(2): e0149216. doi:10.1371/journal.pone.0149216
