# Supplementary material for: Actinobacteria Isolated from an Underground Lake and Moonmilk Speleothem from the Biggest Conglomeratic Karstic Cave in Siberia as Sources of Novel Biologically Active Compounds
Source: PLoS One. 2016 Feb 22;11(2):e0149216. doi: 10.1371/journal.pone.0149216 (PMC4764329; doi:10.1371/journal.pone.0149216)
Supplement: S1 Fig — (PDF) [file pone.0149216.s001.pdf]

SUPPORTING INFORMATION

Actinobacteria isolated from an underground lake and moonmilk speleothem from the biggest conglomeratic Karstic cave in Siberia as sources of novel biologically active compounds

Denis V. Axenov-Gibanov[1*,&], Irina V. Voytsekhovskaya[1,&], Bogdan T. Tokovenko[2], Eugeniy S. Protasov[1], Stanislav V. Gamaiunov[1] , Yuriy V. Rebets[2] , Andriy N. Luzhetskyy[2,3] and Maxim A. Timofeyev[1]

[1]Irkutsk State University, Institute of Biology, Irkutsk, Russia

[2]Helmholtz Institute for Pharmaceutical Research Saarland (HIPS), Saarbrucken, Germany

[3]Universität des Saarlandes – Pharmazeutische Biotechnologie, Saarbrucken, Germany

* Corresponding author:

Denis.axengri@gmail.com

[&]These authors contributed equally to this work.

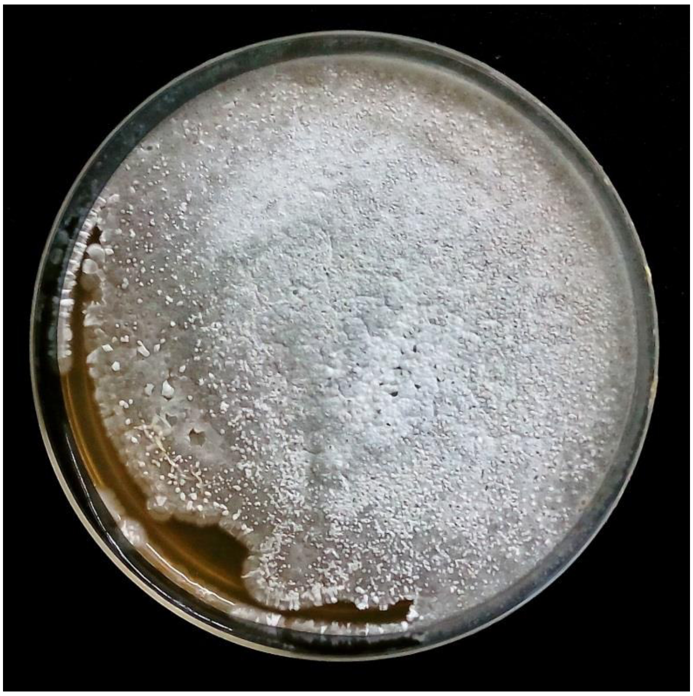

S1 Fig. Photo of strain *Streptomyces sp. IB 2014/I/78-8.*
