# Supplementary material for: Actinobacteria Isolated from an Underground Lake and Moonmilk Speleothem from the Biggest Conglomeratic Karstic Cave in Siberia as Sources of Novel Biologically Active Compounds
Source: PLoS One. 2016 Feb 22;11(2):e0149216. doi: 10.1371/journal.pone.0149216 (PMC4764329; doi:10.1371/journal.pone.0149216)
Supplement: S1 Table — (PDF) [file pone.0149216.s002.pdf]

SUPPORTING INFORMATION

Actinobacteria isolated from an underground lake and moonmilk speleothem from the biggest conglomeratic Karstic cave in Siberia as sources of novel biologically active compounds

Denis V. Axenov-Gibanov[1*,&], Irina V. Voytsekhovskaya[1,&], Bogdan T. Tokovenko[2], Eugeniy S. Protasov[1], Stanislav V. Gamaiunov[1] , Yuriy V. Rebets[2] , Andriy N. Luzhetskyy[2,3] and Maxim A. Timofeyev[1]

[1]Irkutsk State University, Institute of Biology, Irkutsk, Russia

[2]Helmholtz Institute for Pharmaceutical Research Saarland (HIPS), Saarbrucken, Germany

[3]Universität des Saarlandes – Pharmazeutische Biotechnologie, Saarbrucken, Germany

* Corresponding author:

Denis.axengri@gmail.com

[&]These authors contributed equally to this work.

S1 Table. The antimicrobial activity of biomass and culture fluid extracts of cultured actinobacteria strains grown in DNPM/ DNPM^A media.

Strain	DNPM/DNPM^A media																	
	Biomass extract*						Cultural extract*						Biomass-agar extract**					
	B. subtilis	*P. putida*	*E. coli*	*S. cerevisae*	*F. verticillioides*	*C. albicans*	*B. subtilis*	*P. putida*	*E. coli*	*S. cerevisae*	*F. verticillioides*	*C. albicans*	*B. subtilis*	*P. putida*	*E. coli*	*S. cerevisae*	*F. verticillioides*	*C. albicans*
Streptomyces sp. IB 2014 /I /78-8	-	-	-	-	-	-	+(16.3 ±1.5)	+(16.0 ±2)	+(15.3 ±1.5)	-	-	+(10.3 ±0.6) ×	-	-	-	-	-	-
Streptomyces sp. IB 2014 /I /78-1	+(8.3± 0.6) ×	-	-	-	-	-	-	-	-	-	-	-	-	-	-	-	-	-
Nocardia sp. IB 2014 /I /100-5	-	-	-	-	-	-	-	-	-	-	-	-	-	-	-	-	-	-
Nocardia sp. IB 2014 /I /79-1HS	-	-	-	-	-	-	-	-	-	-	-	-	-	-	-	-	-	-
Streptomyces sp. IB 2014 /I /78-12	-	-	-	-	-	-	-	-	-	-	-	-	-	-	-	-	-	-
Streptomyces sp. IB 2014 /I /78-3	-	-	-	-	+(8.3± 0.6)	-	+(12.3 ±0.6)	-	-	-	+(10.3 ±0.6)	-	+(6.3± 0.6)	-	-	+(5.6± 0.6)	+(9.3± 0.6)	+(7.6± 0.6)
Streptomyces sp. IB 2014 /I /78-6	-	-	-	-	-	-	-	-	-	-	-	-	+(13.0 ±2.0)	-	-	-	-	-
Nocardia sp. IB 2014 /I /100-1HS	-	-	-	-	-	-	-	-	-	-	-	-	-	-	-	-	-	-
Streptomyces sp. IB 2014 /I /78-11	-	-	-	-	-	-	-	-	-	-	-	-	-	-	-	-	-	-
Streptomyces sp. IB 2014 /I /78-9	+(11.6 ±0.6)	-	-	-	+(7.6± 0.6) ×	-	-	-	-	-	-	-	-	-	-	-	-	-

* Disc diameter-6mm; ** Disc diameter-4mm

× - Bacteriostatic activity

(Number) – The diameter of inhibition zone (in mm)
