# Supplementary material for: Actinobacteria Isolated from an Underground Lake and Moonmilk Speleothem from the Biggest Conglomeratic Karstic Cave in Siberia as Sources of Novel Biologically Active Compounds
Source: PLoS One. 2016 Feb 22;11(2):e0149216. doi: 10.1371/journal.pone.0149216 (PMC4764329; doi:10.1371/journal.pone.0149216)
Supplement: S2 Table — (PDF) [file pone.0149216.s003.pdf]

SUPPORTING INFORMATION

Actinobacteria isolated from an underground lake and moonmilk speleothem from the biggest conglomeratic Karstic cave in Siberia as sources of novel biologically active compounds

Denis V. Axenov-Gibanov[1*,&], Irina V. Voytsekhovskaya[1,&], Bogdan T. Tokovenko[2], Eugeniy S. Protasov[1], Stanislav V. Gamaiunov[1] , Yuriy V. Rebets[2] , Andriy N. Luzhetskyy[2,3] and Maxim A. Timofeyev[1]

[1]Irkutsk State University, Institute of Biology, Irkutsk, Russia

[2]Helmholtz Institute for Pharmaceutical Research Saarland (HIPS), Saarbrucken, Germany

[3]Universität des Saarlandes – Pharmazeutische Biotechnologie, Saarbrucken, Germany

* Corresponding author:

Denis.axengri@gmail.com

[&]These authors contributed equally to this work.

S2 Table. The antimicrobial activity of biomass and culture fluid extracts of cultured actinobacteria strains grown in NL-19/ MS media.

Strain	NL-19/MS media																	
	Biomass extract**						Cultural extract**						Biomass-agar extract**					
	B. subtilis	*P. putida*	*E. coli*	*S. cerevisae*	*F. verticillioides*	*C. albicans*	*B. subtilis*	*P. putida*	*E. coli*	*S. cerevisae*	*F. verticillioides*	*C. albicans*	*B. subtilis*	*P. putida*	*E. coli*	*S. cerevisae*	*F. verticillioides*	*C. albicans*
Streptomyces sp. IB 2014 /I /78-8	-	-	-	-	-	-	-	-	-	-	-	-	-	-	-	-	-	-
Streptomyces sp. IB 2014 /I /78-1	+(11.3 ±1.5)	-	-	-	-	-	-	-	-	-	-	-	-	-	-	-	-	-
Nocardia sp. IB 2014 /I /100-5	-	-	-	-	-	-	-	-	-	-	-	-	-	-	-	-	-	-
Nocardia sp. IB 2014 /I /79-1HS	-	-	-	-	-	-	-	-	-	-	-	-	-	-	-	-	-	-
Streptomyces sp. IB 2014 /I /78-12	-	-	-	-	-	-	-	-	-	-	-	-	+(10.0 ±2.0)	-	-	-	-	-
Streptomyces sp. IB 2014 /I /78-3	+(5.3± 0.6)	-	-	-	+(9.6± 1.5)	-	+(12.6 ±2.5)	-	-	-	-	-	+(6.3± 0.6)	-	-	-	+(6.6± 1.5)	-
Streptomyces sp. IB 2014 /I /78-6	+(5.6± 0.6)	-	+(14.6 ±1.5) ×	-	-	-	-	-	-	-	-	-	+(5.6± 0.6)	-	-	-	-	-
Nocardia sp. IB 2014 /I /100-1HS	-	-	-	-	-	-	-	-	-	-	-	-	-	-	-	-	-	-
Streptomyces sp. IB 2014 /I /78-11	-	-	-	-	-	-	-	-	-	-	-	-	+(7.3± 2.5)	-	-	-	-	-
Streptomyces sp. IB 2014 /I /78-9	+(12.0 ±2.0)	-	-	-	-	-	-	-	-	-	-	-	-	-	-	-	-	-

** Disc diameter-4mm

× - Bacteriostatic activity

(Number) – The diameter of inhibition zone (in mm)
