# Supplementary material for: Actinobacteria Isolated from an Underground Lake and Moonmilk Speleothem from the Biggest Conglomeratic Karstic Cave in Siberia as Sources of Novel Biologically Active Compounds
Source: PLoS One. 2016 Feb 22;11(2):e0149216. doi: 10.1371/journal.pone.0149216 (PMC4764329; doi:10.1371/journal.pone.0149216)
Supplement: S3 Table — (PDF) [file pone.0149216.s004.pdf]

SUPPORTING INFORMATION

Actinobacteria isolated from an underground lake and moonmilk speleothem from the biggest conglomeratic Karstic cave in Siberia as sources of novel biologically active compounds

Denis V. Axenov-Gibanov[1*,&], Irina V. Voytsekhovskaya[1,&], Bogdan T. Tokovenko[2], Eugeniy S. Protasov[1], Stanislav V. Gamaiunov[1] , Yuriy V. Rebets[2] , Andriy N. Luzhetskyy[2,3] and Maxim A. Timofeyev[1]

[1]Irkutsk State University, Institute of Biology, Irkutsk, Russia

[2]Helmholtz Institute for Pharmaceutical Research Saarland (HIPS), Saarbrucken, Germany

[3]Universität des Saarlandes – Pharmazeutische Biotechnologie, Saarbrucken, Germany

* Corresponding author:

Denis.axengri@gmail.com

[&]These authors contributed equally to this work.

S3 Table. The antimicrobial activity of biomass and culture fluid extracts of cultured actinobacteria strains grown in NL-19/ MS media.

Test culture	Fraction, Minute / Activity																						
	1	2	3	4	5	6	7	8	9	10	11	12	13	14	15	16	17	18	19	20	21	22	23
B. subtilis	-	+(7.6±2.0)×	-	-	-	+(9.3±1.5)×	-	-	-	-	+(6.0±1.0)×	+(11.3±1.5)	-	-	+(6.1±0.2)	-	+(7.3±1.5)	-	-	-	-	-	-
E. coli	-	-	-	-	-	-	-	-	-	-	-	+(11.7±1.5)×	-	-	+(15.0±1.5)×	-	-	-	-	-	-	-	-
P. putida	-	+(7.6±0.6)	-	-	-	+(9.6±1.5)	-	-	-	-	-	-	-	-	-	-	-	-	-	-	-	-	-
C. albicans	-	-	-	-	-	-	-	-	-	-	-	-	-	-	-	-	-	-	+(14.6±1.5)×	+(20.3±2.5)×	+(14.6±2.0)×	+(14.6±3.7)×	+(15.3±2.5)×
S. cerevisae	-	-	-	-	-	-	-	-	-	-	-	-	-	-	-	-	-	-	-	-	-	-	-

(Number) – The diameter of inhibition zone (in mm)

×-Bacteriostatic activity
