# Supplementary material for: Actinobacteria Isolated from an Underground Lake and Moonmilk Speleothem from the Biggest Conglomeratic Karstic Cave in Siberia as Sources of Novel Biologically Active Compounds
Source: PLoS One. 2016 Feb 22;11(2):e0149216. doi: 10.1371/journal.pone.0149216 (PMC4764329; doi:10.1371/journal.pone.0149216)
Supplement: S4 Table — (PDF) [file pone.0149216.s005.pdf]

SUPPORTING INFORMATION

Actinobacteria isolated from an underground lake and moonmilk speleothem from the biggest conglomeratic Karstic cave in Siberia as sources of novel biologically active compounds

Denis V. Axenov-Gibanov[1*,&], Irina V. Voytsekhovskaya[1,&], Bogdan T. Tokovenko[2], Eugeniy S. Protasov[1], Stanislav V. Gamaiunov[1] , Yuriy V. Rebets[2] , Andriy N. Luzhetskyy[2,3] and Maxim A. Timofeyev[1]

[1]Irkutsk State University, Institute of Biology, Irkutsk, Russia

[2]Helmholtz Institute for Pharmaceutical Research Saarland (HIPS), Saarbrucken, Germany

[3]Universität des Saarlandes – Pharmazeutische Biotechnologie, Saarbrucken, Germany

* Corresponding author:

Denis.axengri@gmail.com

[&]These authors contributed equally to this work.

S4 Table. Number and characteristics of main peaks and compounds produced by *Streptomyces sp. IB 2014 /I /78-8.*

N	RT, min	Detected mass, m/z	Accurate mass (M-H+)	UV-maxima, λ	Dictionary of Natural Products					Fraction N
					N hits in DNP	CRC-number	Compound (Accurate mass)	Bio-importance	Source	
1	0,55	419,1532	418,1452	200, 286	0					2
2	0,68	226,1545	225,1465	200, 274	0					2
3	0,68	235,1185	234,1105	200, 270	0					2
4	0,81	254,1607	253,1527	200, 270	0					2
5	0,91	251,1497	250,1417	200, 230, 280	0					2
6	1,31	235,1074	234,0994	200, 230, 270	1	MOS98-O	Sesquichamaenol (AM 234, 16198)		*Cedrelopsis microfoliata*	6
7	1,54	201,1232	200,1152	200, 280	0					6
8	1,67	211,1075	210,0995	200, 277	0					6
9	1,67	228,1338	227,1258	200, 276	0					6
10	1,67	709,3434	708,3354	200, 278	0					6
11	1,76	215,1387	214,1307	200, 260	0					6
12	1,76	482,1809	481,1729	200, 260	0					6
13	1,76	696,312	695,304	200, 260	0					6
14	1,82	285,1342	284,1262	206	0					2
15	2,01	296,1356	295,1276	208, 268	0					6
16	2,5	234,1347	233,1267	200, 220, 275	0					6

17	2,5	261,1228	260,1148	200, 220, 275	0					6,11,12
18	2,8	279,1341	278,1261	200, 270-190, 350	1	OXQ89-J	Monodictysin B (AM 278,115425)	Inhibitor of cytochrome P450 1A activity	*Monodictys putredinis*	2,6
19	2,93	315,1332	314,1252	218, 260, 350	0					11
20	3,9	211,1436	210,1356	218	0					11
21	4,4	245,1279	244,1199	216	0					11
22	4,8	185,0804	184,0724	202, 290	1	MYC84-X	4-Hydroxy-6-(2-hydroxypropyl)-3-methyl-2H-pyran-2-one (AM 184,07356)		*Chaetomium quadrangulatum*	11
23	5	199,1322	198,1242	218, 270, 350	0					11
24	5	225,1482	224,1402	212, 260	0					11
25	5	286,1544	285,1464	218, 270, 350	0					11
26	5,06	373,12790	372,1199	212, 260	2	1) LMV44-L; 2) BFD83-H	1) Quinic acid - 4-O-(3,4,5-Trihydroxybenzoyl) (AM 344,07435); 2) 2′,4′,5-Trihydroxy-7-methoxy-3-prenylflavone (368,12599)	1-; 2-	*1) Quercus stenophylla; 2) Artocarpus integer and Artocarpus elasticus*	11
27	5,11	209,1532	208,1452	218	0					11
28	5,11	227,1638	226,1558	218	0					11
29	5,22	247,1447	246,1367	200-220	0					11
30	5,33	223,1328	222,1248	214, 274	0					12
31	5,33	516,1781	515,1701	214, 274	0					12

32	5,55	217,1438	216,1358	218, 280	1	BBT06-M	1,2,3,4- Tetrahydro-1-methyl-B-carboline; 5-Methoxy (AM 216,126263)	Alkaloid	*Banisteriopsis argentea (Malphigiaceae)*	11,12
33	5,55	433,2802	432,2722	218	0					12
34	5,68	255,0657	254,0577	200, 248, 302	0					12
35	5,74	227,1763	226,1683	190-200	0					11,12
36	5,87	227,1752	226,1672	218, 270	0					11
37	6,08	439,2543	438,2463	218, 265	0					11
38	6,08	704,1836	703,1756	218, 270	0					11
39	6,21	334,1548	333,1468	216, 270	1	NRW95-L	Cyclo(phenylalanyltryptophyl); 3S, 6S-form (AM 333,147727)	Plant growth regulator	*Penicillium sp. and an Aspergillus sp.*	12
40	6,38	555,1504	554,1424	214	1	QGS30-C	Stylissazole B (AM 554,133796)		*marine sponge Stylissa carteri*	11,12
41	6,43	295,1442	294,1362	200, 215	0					12
42	6,91	440,1616	439,1536	220, 280	0					12
43	7,07	698,2234	697,2154	220, 270	0					12
44	7,11	370,1277	369,1197	220, 270	0					11
45	7,15	227,0914	226,0834	217, 264	1	GDJ47-S	2,4-Dihydroxy-3,5,6-trimethylbenzenecarbothioic acid (AM 266,066365)	Antibacterial and antifungal agent	*Mortierella vinacea NRRL 22986*	15
46	7,26	315,1335	314,1255	220, 420	0					11
47	7,26	461,3263	460,3183	220	1	FKY31-S	Ergosta-7,22-diene-3,5,6,9,14-pentol; 6-ketone (AM 460,318875)		*Constit. of Lentinus edodes (shiitake), Hypsizygus marmoreus (bunashimeji), Pleurotus*	15

									ostreatus (oyster mushroom) and Pholiota nameko (nameko)	
48	7,26	957,6644	956,6564	220	0					15
49	7,27	493,3153	492,3073	220	0					12
50	7,27	971,645	970,637	220	0					12
51	7,35	461,3258	460,3178	220, 270	0					12
52	7,35	554,18	553,172	220, 270	1	NPB34-D	Gyrophoric acid; 4-Me ether, L-alanine amide (AM 553,158414)	Inhibitor of diacylglycerol acyltransferase. Claimed antilipaemic agent		12
53	7,35	957,6646	956,6566	220, 270	0					12
54	7,38	259,1544	258,1464	220	0					15
55	7,48	428,3158	427,3078	220, 270	0					12
56	7,48	699,2305	698,2225	220, 270	2	1) ORM27-F; 2) ORM28-G	1) Nepalensinol A (AM 698,2152); 2) Nepalensinol C (AM 698,2152)		*Kobresia nepalensis*	12
57	7,53	225,1491	224,1411	210	0					15
58	7,61	259,154	258,146	220	0					15
59	7,76	359,1481	358,1401	220	3	1)JFS45-E; 2)FYZ17-R; 3) KKJ23-X	1) 6,9-Dihydroxy-4-oxo-2,11(13)-pseudoguaiadien-12,8-olide (AM 358,14164); 2) 15,16-Epoxy-8-hydroxy-3,13(16),14-clerodatriene-18,6:20,12-diolide (AM 358,14164); 3) Triptolide; 14-Ketone (AM 358,14164)	1)-; 2)-; 3) Immunosuppressant	*1) Baileya multiradiata; 2) Jamesoniella autumnalis; 3) Tripterygium wilfordii*	2,6,11

60	7,76	376,1742	375,1662	220	0					11
61	7,76	398,3418	397,3338	220	0					11
62	7,95	227,1643	226,1563	220	0					15
63	7,97	553,1338	552,1258	220	0					11
64	8,11	400,3574	399,3494	220	0					11
65	8,11	759,2384	758,2304	220	0					11
66	8,12	322,1337	321,1257	220, 280	0					15
67	8,23	409,0922	408,0842	222, 270, 400	0					15
68	8,25	398,3417	397,3337	220	0					11
69	8,25	543,3974	542,3894	220	0					11
70	8,25	759,239	758,231	220	0					11
71	8,76	408,1554	407,1474	220, 370	0					15
72	8,77	425,3046	424,2966	222	1	POF13-F	3,20-Diaminopregna 2,14-dien-4-one N20-Me, N3-tigloyl (AM 424,30897)	Alkaloid	*Sarcococca hookeriana*	17,18
73	8,79	357,1332	356,1252	222, 300	0					11
74	9,03	469,3126	468,3046	220	2	1) QNK43-R; 2) BOQ49-Z	1) 18(13->17)-Abeo-3-hydroxy-23-oxolanosta-7,12,24-trien-26-oic acid (AM 468,32396); 2) Stoloniferone B, Ac (AM 468,32396)	1)-:2)-	*1) Abies georgei; 2)Clavularia viridis*	18
75	9,03	1062,5729	1061,565	220	1	QQS86-P	Cyclodysidin D (AM 1061,575714)		*Streptomyces sp. RV15*	18
76	9,2	391,299	390,291	222	0					15
77	9,2	477,3208	476,3128	222	0					15
78	9,2	954,6619	953,6539	222	0					15
79	9,29	354,1445	353,1365	222, 275, 360	0					17
80	9,36	441,2997	440,2917	222	1	QMD43-B	Chaxalactin B. 14-Deoxy (AM 440,29266)		*Streptomyces sp. strain C34*	15

81	9,36	917,6131	916,6051	222	0					15
82	9,42	393,0964	392,0884	222, 400	0					17
83	9,46	387,1797	386,1717	220	0					2
84	9,53	365,1379	364,1299	222	0					17
85	9,68	471,328	470,32	222	4	1) NKF92-E; 2) KJZ92-M; 3) JOP59-H; 4) BOQ52-V	1)2,3-Dihydroxy-5,12-oleanadien-28-oic acid (AM 470,33961); 2) Limatulone (AM 470,33961); 3) 3,4-Seco-4(23),12-oleanadiene-3,29-dioic acid (470,33961); 4) Stoloniferone B; 22,23-Dihydro, Ac (AM 470,33961)	1)-; 2) Ichtyotoxic, feeding inhibitor; 3)-; 4)-	*1) Salvia tomentosa; 2) Collisella limatula; 3) Maytenus undata; 4) Clavularia viridis*	15
86	9,68	797,4661	796,4581	222	1	CPY02-M	3,6,16-Trihydroxycycloart-25-en-24-one; (3β,6α,16β)-form, 3,16-Di-O-B-glucopyranoside (AM 796,46091)		*Astragalus trigonus*	15
87	9,68	913,5138	912,5058	222	0					15
88	9,77	423,2897	422,2817	222	0					17
89	10,03	425,3047	424,2967	222	0					17,18
90	10,03	425,3051	424,2971	222	0					15
91	10,09	440,354	439,346	222	0					12
92	10,13	391,2994	390,2914	222	0					15
93	10,13	906,6805	905,6725	222	0					15
94	10,16	443,3156	442,3076	222	0					17
95	10,16	921,6459	920,6379	222	0					17
96	10,16	1381,967	1380,959	222	0					17
97	10,2	393,0966	392,0886	222, 400	0					18
98	10,31	459,3109	458,3029	222	3	1) PVJ60-I; 2)	1) 22-Hydroxy-25-oxo-16-scalaren-24-oic acid; 18B-		*1) Smenospongia*	17

						GZY73-S; 3) OFK01-D	form. 22Ac, Me ester (AM 458,303225); 2) 1,3,27-Trihydroxywitha-5,24-dienolide (AM 458,303225); 3) 1,3,7-Trihydroxywitha-5,24-dienolide (458,303225).		*sp.; 2) Physalis pubescens, Withania somnifera; 3)-*	
99	10,37	391,2996	390,2916	222	0					18
100	10,37	835,6221	834,6141	222	0					18
101	10,44	393,3158	392,3078	218	0					17
102	10,44	411,3266	410,3186	218	0					17
103	10,64	427,3202	426,3122	222	2	1) HGK04-O; 2) HGK05-P	1) Stoloniferone A (AM 426,313395); 2) Stoloniferone B (426,313395)	1) Cytotoxin; 2) Cytotoxin	*1) Clavularia viridis; 2) Clavularia viridis*	18
104	10,64	634,3018	633,2938	222	0					18
105	10,64	906,6789	905,6709	222	0					18
106	10,94	445,3318	444,3238	222	0					18
107	11,07	423,28940	422,2814	222	0					18
108	11,07	656,39760	655,3896	222	0					18
109	11,8	520,26020	519,2522	222, 280	0					18
110	15,95	546,48750	545,4795	224	0					18
111	18,39	391,2837	390,2757	224	2	1) PVB60-U; 2)PVK06-B	1) Aculeatin F (390,27701); 2)Aculeatin F; 4,6-Diepimer (AM 39,27701)		*Amomum aculeatum*	2,6
112	18,39	803,542	802,534	224	0					2
113	18,41	1097,6221	1096,614	224	0					17,18
114	18,41	1114,6514	1113,643	224	0					17,18
115	18,41	1259,637	1258,629	224	0					17,18
116	19,04	1128,6698	1127,662	224	0					15
117	19,05	1128,6739	1127,666	224	0					17,18

118	19,06	702,2126	701,2046	224	0					2
119	19,06	1128,6664	1127,658	224	0					2
120	19,67	1142,6843	1141,676	224	0					17,18
121	19,68	1259,6843	1258,676	224	0					18
122	20,34	663,4523	662,4443	224	0					2
